# The role of INTERCheckWEB digital innovation in supporting polytherapy management

**DOI:** 10.1038/s41598-023-32844-6

**Published:** 2023-04-04

**Authors:** Emanuela Foglia, Lucrezia Ferrario, Elisabetta Garagiola, Federica Asperti, Antonino Mazzone, Federico Gatti, Luca Varalli, Cristina Ponsiglione, Lorella Cannavacciuolo

**Affiliations:** 1 LIUC Business School, LIUC- University Cattaneo, Healthcare Datascience LAB, Corso Matteotti 22, 21053 Castellanza, Varese Italy; 2 ASST Ovest Milanese Hospital, Legnano, Milano Italy; 3 Department of Industrial Engineering, University of Naples Federico II, Naples, Italy

**Keywords:** Health care, Medical research

## Abstract

The study aims at defining the factors affecting the clinicians’ decision of changing or confirming the treatment options for frail patients in polytherapy, supporting prescribing patterns, thus also figuring out if the inclination of the clinicians towards digital solutions (INTERCheckWEB) and specific guidelines, could play a role in their decision. A literature review was performed, revealing the main individual, organizational and decisional factors, impacting on the clinicians’ propensity to change the current patients’ therapy: the clinician perceptions of support in case of clinical guidelines use or INTERCheckWEB use were studied. A qualitative approach was implemented, and thirty-five clinicians completed a questionnaire, aimed at evaluating fifteen different clinical cases, defining if they would change the patient’s current therapy depending on the level of information received. Three methodological approaches were implemented. (1) Bivariate correlations to test the relationships between variables. (2) Hierarchical sequential linear regression model to define the predictors of the clinician propensity to change therapy. (3) Fuzzy Qualitative Comparative Analysis—fsQCA, to figure out the combination of variables leading to the outcome. Patient’s age and autonomy (*p* value = 0.000), as well as clinician’s perception regarding IT ease of use (*p* value = 0.043) and seniority (*p* value = 0.009), number of drugs assumed by the patients (*p* value = 0.000) and number of concomitant diseases (*p* value = 0.000) are factors influencing a potential change in the current therapy. The fsQCA-crisp confirms that the clinical conditions of the patients are the driving factors that prompt the clinicians towards a therapy change.

## Introduction

Clinical decision-making represents “a complex action involving information processing, evaluation of evidence, and application of relevant knowledge to select the appropriate interventions, that provide high-quality care and reduce risk of patient harm”^1^. In this view, the main objective of any clinical decision-making process is to define an informed judgement about the diagnostic and treatment pathways necessary for guaranteeing patients’ health, thus being the key-point of healthcare policy and medical practice^2^.

Since health outcomes are probabilistic, most decisions are made under conditions of uncertainty. In this regard, the presence of comorbidities and multiple chronic diseases, and the related prescription of complex medications, as well as concerns in therapies changes, present many professional challenges in the clinical prescribing decision-making process. This topic acquires a strategic relevance, since older adults have a higher prevalence of multiple chronic health conditions, for which multiple medications are recommended^3^. Consequently, multiple medications’ use (i.e. polypharmacy) could be associated with adverse outcomes^4^, such as drug-drug interactions (DDIs) or drug-related adverse events, as well as a higher risk of falls in the elderly^5^, a greater mortality and a higher level of complications, when hospitalized^6,7^. By incorporating a comprehensive prescribing decision-making process that considers a patient's complete medication regimen, healthcare professionals can reduce the risk of DDIs and improve patient outcomes. This process includes identifying potential DDIs, evaluating the risks and benefits of each medication, and selecting the most appropriate medication or combination of medications^8–11^. These factors could significantly impact in the situation to manage patients affected by COVID-19: these patients will often be prescribed additional medications to treat their COVID-19 along with their regular medication regimen, which will further increase the risk of experiencing DDIs^12^, due to the intrinsic nature of the anti-COVID-19 therapies. For example, some of the medications used to treat COVID-19, such as hydroxychloroquine, azithromycin, and remdesivir, have been associated with QT interval (referring to the length of time between the start of the Q-wave and the end of the T-wave, useful to measure electrical properties of the heart) prolongation, which can increase the risk of serious arrhythmias when used in combination with other medications that also have the same effect, prolonging the QT interval^13,14^. Additionally, some medications used to treat COVID-19 may be metabolized in the liver by the same enzymes, which can lead to drug interactions, affecting the efficacy or safety of the medications^15^. As such, the management of their clinical condition is becoming an urgent priority, to prevent immediate and long-term adverse effects, unexpected outcomes, and mortality^14–17^.

In general terms, approximately half of the elderly population is prescribed a non-suitable or non-desirable drug, with a higher incidence in poly-pathological patients: in some cases, the prevalence of potentially inappropriate medications can reach 71%, in frail elderly people^18^.

According to the above, difficulties in the management of such patients often occur in the clinical practice, since clinicians should monitor also drugs prescribed by other healthcare professionals. Clinicians, especially those practicing in primary care, geriatrics, mental health care, and emergency departments, frequently encounter several challenges in caring for patients with multiple chronic conditions^19^.

Hence, this situation puts in evidence how a reduction of potentially inappropriate prescriptions/medications for elderly patients in polypharmacy, represents a relevant topic, requiring an in-depth analysis.

Any methods of facilitating guidance on managing patients with polypharmacy, through the development and application of ‘risk prediction tools’ for quantifying the risk of adverse drug reactions, acquire a strategic relevance^20,21^. In this view, clinicians may benefit from considering evidence-based recommendations of drug use to preserve patient safety, worldwide. According to this consideration, the prescription decision-making process could leverage on digital solutions able to guide the clinicians in making an appropriate prescription for elderly patients in polytherapy, thus checking the potential DDIs and guiding whether or not to prescribe a drug reported that Computerized Prescription Support Systems (CPSS) might change healthcare provider behaviour, improving clinicians’ performance, and reducing DDIs and the number of inappropriate prescriptions^22,23^. As such, CPSS can help identify potential DDIs and provide alerts to prescribers, thus resulting in a reduction in the risk to develop any adverse events or to experience hospitalization, enhancing the overall patients’ outcomes^24,25^.

Although the availability of digital innovations to support the decision-making in polypharmacy, their use is not diffused, as highlighted by the review of Arcopinto and colleagues, revealing the absence, at least in the Italian context, of key policies and procedures for polypharmacy management in older adults, thus also suggesting the use of ICT-based approach in addressing these issues^26^. Furthermore, little is known concerning the factors that could prompt the clinicians, to leverage on this digital solution, to make such prescriptions^27^.

Digital solutions and guidelines can be assimilated to rules that could reduce the clinicians’ discretionary judgment, based on own reasoning. Thus, the prescription decision should be considered as the result of a complex decision-making process where the diagnosis is made after an interpretation of information and related choices^28,29^. Individual characteristics of any decision-maker and the complexity of task could affect the individual behaviour, in making decisions^30–32^. Among the individual characteristic, the attitude of clinicians to consider the suggestions given by digital solutions and guidelines would be considered.

Moving on from these premises, the present study aims at shedding a light on factors affecting the clinicians’ decision of changing or confirming the treatment options in frail patients in polytherapy, supporting prescribing patterns, thus also figuring out if the inclination of the clinicians towards digital solutions and specific guidelines could play a role in their decision.

In particular, the study focused on one of the most outstanding digital innovations: INTERCheckWEB, a CPSS for helping clinicians during their routinely clinical practice, improving the appropriateness of drugs prescription^33^. INTERCheckWEB is a free and user-friendly interface, enabling the clinician gather information on specific drugs or DDIs and facilitating the evaluation of any possible therapy switch or change. INTERCheckWEB stores useful information concerning potentially inappropriate medications, anticholinergic burden, and dose adjustment, in case of renal disease and modality for drug withdrawals, thus relying on the most recent versions “Beers”, and “START and STOPP” criteria^34,35^.

Coherently with these premises, the study addressed the following research questions (RQs).

- RQ1) “Which are the factors determining the change in the treatment options, in frail patients in polytherapy?”.
- RQ2) “How the combination of clinicians’ attitudes towards specific information deriving from CPSS “INTERCheckWEB”, guidelines and other factors affect the change in the treatment options in frail patients in polytherapy?”.

### The conceptual research framework

To answer the above RQs, the conceptual framework assumes that the prescription decision process is a dynamic action, based on the interpretation of new available information, that could prompt the decision maker to revise the accuracy of his/her decisions^28^. The decision makers exhibit different strategies in selecting and making sense to information to reach an appropriate decision, as the information could be perceived differently^36–38^. Individual characteristics, organizational context and decisional tasks are the factors that could influence clinicians in making decisions^30^. On the one hand, individual characteristics concern the personal capability to interpretate new information and identify new solutions through a learning and adaptation process to situations^28^. Usually these factors refer to age, socio-economic roots, education, career, and working experiences^39^. On the other hand, the decisional tasks include the characteristics of the problem and its complexity, such as information overload, number of alternatives, time pressure, and so on^40–42^. In addition, factors related to the organizational context considers that the individuals, belonging to the same organization, share the rules of thumbs, spreading through a social process of influence and imitation, that could affect the individual decision-making^43,44^.

In this study, the individual factors also include the behaviour of clinicians in using guidelines and technologies to measure how the effect of a different inclination of clinicians towards these sources of knowledge cold influence their final prescription decision, to change the therapies. Concerning the behaviour of clinicians towards the technology, the constructs of the Technological Acceptance Model – TAM were assumed^45^. According to TAM, users’ adoption of digital technologies is determined by perceived usefulness and perceived ease of use, identifying the clinicians’ attitudes toward the investigated technology^45–47^. The organizational factors are not relevant for this study: all the organizations promote the use of INTERCheckWEB, and therefore the use of this platform depends exclusively on clinicians’ intentions to use this tool, in managing frail patients with comorbidities. Since INTERCheckWEB is a free and open access tool, usable on any type of computer, thus being highly scalable, and grounds its use only on the Internet connection presence (also with a smartphone application in case of absence, in the hospital wards, of a personal computer or an Internet connection), no organizational problems would emerge in its routinely adoption.

Moving from this essential premise, the study focused the attention on task complexity and on the individual factors impacting on the clinicians’ choice to change patients’ therapies, that is a totally individualistic topic, without any organizational barrier, implication, or limit.

In summary, the factors, that could impact on the clinicians’ choice to change the patients’ current therapy, in the management of elderly and poly-pathological patients, concern these two elements.

- Individual variables: seniority, clinicians’ behaviour towards guidelines and clinical protocols, clinicians’ behaviour towards digital technologies (such as INTERcheckWEB), split in the two constructs of “usefulness” and “ease of use”.
- Decisional tasks: patients’ polypharmacy/polytherapy, patients’ comorbidities, quality of available patients’ data.

Based on these factors, the research framework identifies the following hypotheses, analysing the factors in the situation of a clinical decision-making process.

#### Clinicians’ behaviour towards guidelines and clinical protocols

Clinical guidelines have been upheld as an essential part of quality medical practice for several decades, and they have been systematically developed to assist healthcare professionals and patients in making treatment decisions^48^. Despite the growing number of guidelines, their use in practice is slow and complex, and often not applied at all^49^. In general terms, clinicians are prone to use guidelines and recommendations: they should be aware of a guideline and need to have some knowledge of their contents. Clinicians’ knowledge and reliability of the guidelines influence attitudes, and attitudes affect clinical practice behavior^50^. In the current clinical setting, the more the clinician is aware to the quality of guidelines and clinical protocols information, the more she/he is influenced in changing the current clinical practice.

According to the above, the following hypotheses were developed.

- *Hp 1* The negative clinicians’ behaviour towards guidelines and clinical protocols has a negative impact on the clinicians’ propensity to change the current patients’ therapy.
- *Hp 2* The positive clinicians’ behaviour towards guidelines and clinical protocols has a positive impact on the clinicians’ propensity to change the current patients’ therapy.

#### INTERCheckWEB usefulness

In the routine practice, the clinicians tend to use any innovative technology, such as INTERCheckWEB, only if they believe IT could improve daily activities and the proper use of the tool itself, aiming at the best quality and safety, for the patients^46,51^. Hence the IT usefulness perceptions increase, the more the probability that this tool will help the clinician to make the most appropriate choice^45,46^.

- *HP 3* INTERCheckWEB perceived usefulness has a positive impact on the clinicians’ propensity to change the current patients’ therapy.

#### INTERCheckWEB ease of use

INTERCheckWEB ease of use should be defined as the lack of effort in understanding its functioning, thus leading the clinician to use this tool in the routinely clinical practice, in his/her decision-making process concerning the patients’ therapeutic treatment^45,46^.

In particular, the greater the INTERCheckWEB ease of use, the greater the willingness to make different choice given the tool suggestions, concerning the development of potential DDIs.

- *HP 4* INTERCheckWEB perceived ease of use has a positive impact on the clinicians’ propensity to change the current patients’ therapy.

#### Clinicians’ seniority

Clinical performance has been proven to be associated with clinicians’ own experience and seniority^43,44^. As such, literature stated that clinicians with a longer seniority are more likely to use guidelines to address their clinical practice^54^. Additionally, the more the clinicians know about guidelines and protocols, the more the clinicians learn about the potential development of DDIs or adverse events, leading to a possible change of the current patient’s therapy^55^. As reported by Smith and colleagues^30^, clinical decision-making process is affected by several factors, such as clinicians’ experience and ability, in making decisions regarding a given pharmacological treatment.

- *HP 5* The clinicians’ seniority has a positive impact on the clinicians’ propensity to change the current patients’ therapy.

#### Patients’ polypharmacy

Polypharmacy refers to the use of multiple medications. Specific combination of various drugs administered to a single patient could result in an interaction^56^: as the number of medications increases, the potential for drugs’ interaction increases. In this view, the number of drugs administered could be an interesting topic to change the patients’ current therapy, by stopping medication and thereby improving the patients’ health^57,58^.

- *HP 6 *Polytherapy has a positive impact on the clinicians’ propensity to change the current patients’ therapy.

#### Comorbidities

Patients suffering from multiple and concomitant diseases present a higher possibility to develop DDIs^57,59^, thus leading the clinicians to deprescribing activities. For clinicians, multiple chronic conditions may serve as competing demands on time, or act as barriers that may influence access to drug, and decisions regarding treatments^60^. Literature stated that multi-morbidity would suggest the clinicians to change the current therapy, to avoid the development of adverse events^57^.

- *HP 7* Patient concomitant disease has a positive impact on the clinicians’ propensity to change the current patients’ therapy.

In addition to the direct relationships between the above-mentioned independent variables and the clinical propensity to change the current patients’ therapy (playing, instead, the role of dependent variable), the proposed framework also includes three moderators (two of them acting also as independent variables): patients’ polypharmacy, quality of available patients’ data, and clinicians’ seniority.

Concerning patients’ polypharmacy, Vehoof and colleagues stated that the higher the number of concomitant diseases of a patient, the higher the number of drugs that the patients should assume for the satisfaction of his/her healthcare needs^61^. In this view, the number of drugs is assumed to strengthen the relationship between patients’ comorbidity and clinician’s propensity to change the current therapy.

- *HP 8* Polypharmacy positively moderates the relationship between patients’ comorbidity and the clinicians’ propensity to change the current patients’ therapy.

The quality of available patients’ data could be defined as the quality, appropriateness, and the presence of a large amount of detailed and complete information, recorded for a given patient. The lack of clinical information concerning a patient, or the absence of some anamnestic or comorbidities data, or the presence of potential errors in their collection, do not allow the clinician to identify a correct pharmacological treatment^62^. According to the above, the following hypothesis concerning the moderator effect is displayed.

- *HP 9* The quality of available patients’ data positively moderates the relationship between polytherapy and the clinicians’ propensity to change the current patients’ therapy.

Since, as previously described, no organizational factors could limit INTERCheckWEB potential use, we focused the attention on specific individual variables, that could act as moderators in the relationship among variables. Clinicians’ seniority, in terms of increasing experience over time, would strengthen the relationship among (i) the positive clinicians’ behavior towards guidelines and clinical protocols; *ii)* INTERCheckWEB ease of use; and (ii) INTERCheckWEB perceived usefulness, and the clinicians’ propensity to change the current therapy.

On the one hand, the higher the clinicians’ seniority, the greater his/her knowledge concerning guidelines and protocols, with relevant consequences in terms of therapy changes^30^. On the other hand, clinicians with a greater experience could present a low-level IT systems knowledge, thus negatively influencing INTERCheckWEB ease of use, and usefulness^63,64^.

- *HP 10* Clinicians’ seniority positively moderates the relationship between positive clinicians’ behavior towards guidelines and clinical protocols and clinicians’ propensity to change the current patients’ therapy.
- *HP 11* Clinicians’ seniority negatively moderates the relationship between INTERChekWEB ease of use and clinicians’ propensity to change the current patients’ therapy.
- *HP 12* Clinicians’ seniority negatively moderates the relationship between INTERChekWEB usefulness and clinicians’ propensity to change the current patients’ therapy.

Furthermore, for the creation of a comprehensive framework, the following set of control variables was assumed: patient’s age, patient’s autonomy, and patient’s body max index (BMI).

**Figure 1:**
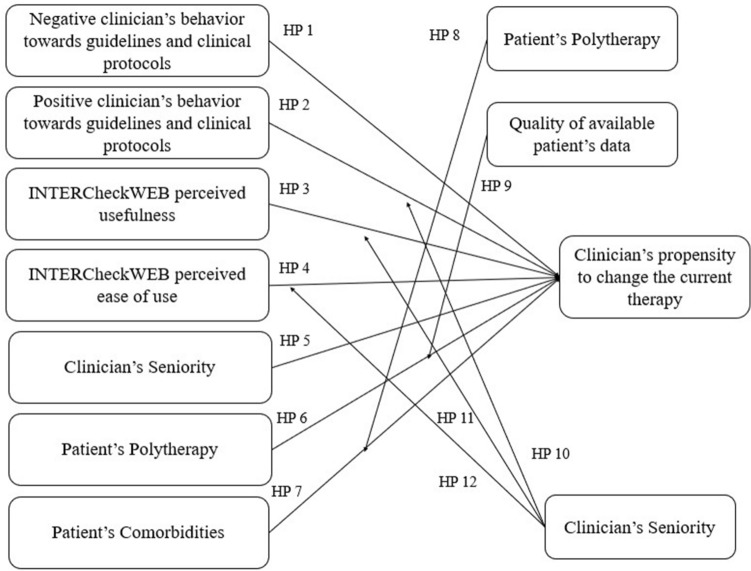
Framework.

A synthesis of the research framework is proposed in Fig. 1.

## Methods

### Data collection

The data collection of the study was approved by the ASST Ovest Milanese committee being the Healthcare Directorate of the hospitals involved, according to the study protocol number 5135 (Class 03.08.01) dated 8th February 2019.

After having received the approval, the study involved head physicians and clinicians (N = 35) referring to the Internal Medicine wards, of four medium size hospitals, located in Northern Italy. In June 2019, a questionnaire was created aimed at collecting not only clinicians’ demographic information, such as age, gender, seniority, but also their behaviour towards guidelines and protocols, as well as the INTERCheckWEB ease of use and usefulness, according to specific constructs validated by literature^45–47^. In addition, the questionnaire presented 15 clinical cases of patients in polypharmacy and suffering from multiple diseases. This was useful to define if in case of specific information derived from INTERCheckWEB, they would have changed the patient’s current therapy, during an Internal Medicine hospitalization. Information regarding the clinical cases concern the following factors: patients’ BMI, age, autonomy, number of concomitant diseases, number of drugs assumed, definition and number of the potential interactions that could occur before INTERCheckWEB support.

At first, the questionnaire was validated during a specific focus group^65^—composed of 6 clinicians and 2 researchers—that also defined both the clinicians to be involved in the study and the clinical cases to be investigated.

On the one hand, the clinicians involved in the study were representative of the experts working in the Internal Medicine Wards, but usually taking rotations also in the Emergency Departments, and managing patients with therapeutic strategies prescribed by primary care professionals or general practitioners. These professionals represent the best unit of analysis, because being devoted in the management of frail patients, with multiple chronic diseases and taking several medications, thus being the professionals more prone to make clinical decisions under stressful and emergency conditions^59,66^. On the other hand, the clinical cases were selected based on their clinical condition in terms of number of concomitant diseases and number of drugs administered to hospitalized patients^67^, assuming the time-horizon of 60 days before the institution of the focus groups.

After the selection of clinicians and clinical cases, the questionnaire was completed by all the professionals involved in the study, through a structured interview. Once the clinicians completed the first part, answering the questions related to their behaviour towards guidelines, protocols, and digital technologies, and after having carefully read each clinical case, the results derived from INTERCheckWEB, in terms of development of potential interactions and adverse events, were shown to the clinicians to understand if this additional information would determine a possible change in therapies prescribed. This data activates the interpretation process of the clinicians and could lead the professionals to cultivate a new decision on therapies, changing them, with respect to the previous decision made without the digital platform support.

According to the above, the clinician’s propensity to change the current patient therapy was measured as the number of ceasing or additional drugs that could be prescribed to the patients, in comparison with the current situation of drugs prescribed.

### Data analysis

Data derived from the questionnaire were first analysed considering descriptive statistics. Preliminary analyses were performed to ensure no violation of the assumptions of normality, linearity, and homoscedasticity.

Furthermore, data were assessed by means of three methodological approaches, to achieve the study objective and answer to the above-mentioned research questions.

Firstly, we perform data analysis according to the following inferential analyses, using IBM SPSS Statistics software (version 27).

- Relationships between variables, were investigated to test the existence of correlations among them.
- A hierarchical sequential linear regression model was implemented to define the predictors of the clinicians’ choice to change the current therapy, establishing the impacts of independent variables and moderators respectively, as previously defined. The regression models were introduced to study the impact of the single variables on the clinicians’ choice to modify the current patients’ therapy. Three models were thus defined as follows.
  - *Model 1* composed of only the control variables (patient’s age, patient’s autonomy, and patient’s BMI).
  - *Model 2* composed of Model 1, with the inclusion of the independent variables (clinicians’ behaviours towards guidelines and clinical protocols, InterCheckWEB usefulness and ease of use, clinicians’ seniority, patient’s polypharmacy, comorbidities).
  - *Model 3* composed of Model 1 and Model 2, with the inclusion of the moderator variables (the effects of patients’ polypharmacy, quality of available patients’ data, and clinicians’ seniority on the dependent variables).

The Adjusted R^2^ was examined, verifying the explanatory power of each model.

Then, a Qualitative Comparative Analysis—QCA^68,69^ was applied to gather more finer-grained details about the underlying antecedents of clinicians’ decision-making. QCA complements the analysis of regression models inasmuch it can reveal asymmetries and multiple pathways, hidden in the data^70^. QCA is a comparative case-oriented, based on complexity theory, aimed at identifying the configurations of causally relevant conditions linked to the outcome under investigation^71^. The basis of QCA’s configurational approach is the analysis of sufficient and necessary causes to produce an outcome: a condition is necessary if it is present in all configurations of the outcome, which means that the outcome cannot be achieved without the presence of this condition^72^. One condition will suffice if a particular outcome emerges whenever the condition is present^72^. However, there might be other conditions that lead to the same result; in other words, there could be multiple sufficient causes^72^. QCA operates at the level of observed cases and considers each case as a holistic configuration of conditions^73^. The conditions are the relevant variables that, in our investigated phenomenon, are inferred by the literature and exanimated in the regression model. A QCA fuzzy (fsQCA) approach was adopted to figure out the combinations of these variables that could lead to the outcome (clinician’s propensity to change the current therapy), using the software fsQCA version 3.1b.

The application of fsQCA requires the calibration of the variables. The calibration aims at transforming the value of the variables into a fuzzy set, ranging from 1 (full membership of variable to the configuration) to 0 (full no-membership of the variable to the configuration). A value of membership between these values [1, 0] means that this variable belongs to the configuration with a certain degree, not completely. It occurs that the variable can assume only the value 1 or the value 0. In this case the QCA variable is crisp. The calibration requires the definition of the thresholds that gives the level of membership in the fuzzy set. These thresholds could be obtained through a direct or indirect method, depending on underlying theory and knowledge about the phenomenon under investigation^74^.

A direct method was applied if the researchers establish the thresholds based on theory or knowledge. In the indirect method, according to the distribution of the data, the full membership, intermediate membership, and no-membership thresholds were defined respectively equal to 0.95, 0.50, 0.05 percentiles with a normal distribution^72^ or 0.8, 0.5 and 0.2 percentiles with a skewed distribution^75^.

The calibration output is available on request. Based on the calibration, the fsQCA builds the truth table, which groups empirical cases since they show the presence or absence of the outcome. Defining a consistence threshold equal to 0.80 and a frequency threshold equal to 3 because our sample is larger than 150 cases (presenting 525 observations)^76,77^, the consistent and empirically relevant patterns (causal configurations of conditions) pertaining to the outcome were obtained. According to the above fsQCA produces both consistence and coverage, with values ranging from 0 to 1. On the one hand, the consistence measures whether a configuration produce the outcome in the real data, whereas the coverage indicates how many cases are covered by such configuration^78^.

## Results

### The sample under assessment

The sample of clinicians (N = 35) was mostly composed of females (63%). The average age of the clinicians involved was 45.23 years old, with a working experience of 16.14 years.

Concerning the clinical cases studied (N = 15) and evaluated from the clinicians involved, the sample was mostly composed of females (60%), with an average age of 78.41 years (range: 65–93). The patients at the hospital admissions assumed on average 9 different drugs (range: 5–12).

### Results from the inferential analysis

Table 1 depicts all the potential relationships between variables, identifying the positive or negative impact of clinicians’ behaviour towards guidelines and clinical protocols, INTERCheckWEB perceived usefulness and ease of use, clinician’s seniority, patient’s polytherapy and patient’s comorbidities, on the clinician’s propensity to change the current patient’s therapy.

**Table 1:** Relationships among variables.

	1	2	3	4	5	6	7	8	9	10	11	12	13	14	15	16
Clinician's propensity to change the current therapy (1)	1															
Patient’s age (2)	.110^*^	1														
Patient’s autonomy (3)	− .034	.073	1													
Patient’s BMI (4)	.548^**^	.080	− .045	1												
Negative clinician’s behaviour towards guidelines and clinical protocols (5)	− .098^*^	.000	.000	.000	1											
Positive clinician’s behaviour towards guidelines and clinical protocols (6)	− .026	.000	.000	.000	.000	1										
INTERcheckWEB perceived usefulness (7)	.041	.000	.000	.000	− .235^**^	− .003	1									
INTERcheckWEB perceived ease of use (8)	.082	.000	.000	.000	− .281^**^	.003	.825^**^	1								
Clinician’s seniority (9)	.084	.000	.000	.000	− .208^**^	− .032	− .094^*^	− .137^**^	1							
Patient’s polytherapy (10)	.579^**^	.191^**^	− .486^**^	.568^**^	.000	.000	.000	.000	.000	1						
Patient’s comorbidities (11)	.211^**^	.283^**^	− .245^**^	.596^**^	.000	.000	.000	.000	.000	.513^**^	1					
Patient’s polytherapy X patient’s comorbidities (12)	.511^**^	.216^**^	− .432^**^	.688^**^	.000	.000	.000	.000	.000	.931^**^	.782^**^	1				
Quality of available patient’s data X patient’s polytherapy (13)	.520^**^	.295^**^	− .416^**^	.613^**^	.000	.000	.000	.000	.000	.936^**^	.720^**^	.959^**^	1			
Clinician’s seniority X positive clinician’s behaviour towards guidelines and clinical protocols (14)	− .005	.000	.000	.000	.097^*^	.883^**^	− .005	− .052	.010	.000	.000	.000	.000	1		
Clinician’s seniority X INTERcheckWEB perceived usefulness (15)	.014	.000	.000	.000	− .222^**^	− .006	.889^**^	.791^**^	− .015	.000	.000	0.000	.000	− .087^*^	1	
Clinician’s seniority X INTERcheckWEB perceived ease of use (16)	.038	.000	.000	.000	− .213^**^	− .050	.713^**^	.892^**^	− .092^*^	.000	.000	.000	.000	− .161^**^	.875^**^	1

*p-value > 0.05; ** p-value > 0.01.

It emerged that a higher clinician’s propensity to change the current patient’s therapy was strictly related to patient’s age (β = 0.110, *p* value = 0.011) and BMI (β = 0.548, *p* value = 0.000). Furthermore, a significant negative relationship was reported between the negative behaviour towards guidelines and clinical protocols and the propensity to change the current therapy (β = − 0.098, *p* value = 0.024). A clinician is more likely to change therapy if the patient assumed a greater number of drugs (β = 0.211, *p* value = 0.000), as if the patient suffered from a higher number of concomitant diseases (β = 0.511, *p* value = 0.000). The moderator variables related to patient’s data quality and polytherapy, impacted on the clinical choice to change therapy.

Focusing on the relationships among the control variables and the independent variables, the following considerations emerged.

- Patient’s age was strictly related to the development of concomitant diseases (β = 0.283, *p* value = 0.000) and to polytherapy (β = 0.191, *p* value = 0.000). In particular, the older the patient, the greater the number of comorbidities, and consequently the number of drugs assumed.
- A higher BMI was reported in patients suffering from multiple diseases (β = 0.568, *p* value = 0.000) and assuming a greater number of drugs (β = 0.596, *p* value = 0.000).
- A low-level of autonomy emerged in patients with a higher number of comorbidities (β = − 0.245, *p* value = 0.000) and drugs (β = − 0.486, *p* value = 0.000).
- Clinician’s seniority presented a negative relationship with the negative clinician behavior towards guidelines and protocols (β = − 0.208, *p* value = 0.000), as well as lower perceptions of INTERCheckWEB usefulness (β = − 0.094, *p* value = 0.031) and ease of use (β = − 0.137, *p* value = 0.002).

The regression model (Table 2) demonstrated that younger patient’s age (β = − 0.073, *p* value = 0.048), autonomy (β = 0.303, *p* value = 0.000) and BMI (β = 0.505, *p* value = 0.000), as well as clinician’s perception with regard to IT ease of use (β = 0.298, *p* value = 0.043) and clinicians’ seniority (β = 0.087, *p* value = 0.009), number of drugs assumed by the patients (β = 0.541, *p* value = 0.000) and number of concomitant diseases (β = 0.302, *p* value = 0.000) are factors influencing a potential change in the current therapy.

**Table 2:** Regression models.

	Model 1	Model 2	Model 3
**Control variables**			
Patient’s age	0.068*	0.031	− 0.073*
Patient’s autonomy	− 0.015	0.203*	0.303*
Patient’s BMI	0.542*	0.383*	0.505*
**Independent variables**			
Negative clinician’s behaviour towards guidelines and clinical protocols		− 0.060	− 0.061
Positive clinician’s behaviour towards guidelines and clinical protocols		− 0.024	− 0.104
INTERcheckWEB perceived usefulness		− 0.089	− 0.084
INTERcheckWEB perceived ease of use		0.150*	0.298*
Clinician’s seniority		0.083*	0.087*
Patient’s polytherapy		0.599*	0.541*
Patient’s comorbidities		− 0.283*	0.302*
**Moderators**			
Patient’s polytherapy X patient’s comorbidities			− 0.370*
Quality of available patient’s data X patient’s polytherapy			− 0.661*
Clinician’s seniority X positive clinician’s behavior towards guidelines and clinical protocols			0.083
Clinician’s seniority X INTERcheckWEB perceived usefulness			− 0.035
Clinician’s seniority X INTERcheckWEB perceived ease of use			− 0.134
R^2^	0.305	0.516	0.550
Adjusted R^2^	0.301	0.506	0.537
F value	76.236*	54.751*	41.555*
Δ R^2^	0.305	0.211	0.035
F(ΔR^2^)	76.236*	31.954*	7.859*

The moderator effects of both the data quality and polypharmacy (β = -0.661, *p* value = 0.001), and the number of drugs and the number of comorbidities (β =  − 0.370, *p* value = 0.000) were confirmed.

The above aspects explained the 53.7% of the clinician’s choice variance, to modify the prescription, reducing the number of treatments to be administered to the patients.

### Results from the QcA

QCA allows to analyse the outcome (clinician’s propensity to change the current therapy) considering also possible combinations of the variables. Based on a consistence threshold equal to 0.80 and a frequency threshold equal to 3 inasmuch our sample is larger than 150 cases^76,77^, 11 consistent and empirically relevant patterns (configurations of the variables) pertaining to the outcome (clinician’s propensity to change the current therapy) were obtained. Figure 1 shows the configurations emerged from the fsQCA analysis.

The configurations belong to the intermediate solution that include the parsimonious solution. In this way, we can keep account both the core variables and the peripheral ones. The core variables have a strong causal relation with the output, instead the peripheral ones have a weak causal relation. Usually, the presence of a variable in a given configuration is shown with a black circle, the negation of the variable with a crossed-out circle, the blank space indicates that the variable doesn’t care.

The fsQCA results exhibit the configuration 2 as the pattern that present both a high value of consistency (0.870273) and the highest value of coverage (0.12). This configuration considers that the outcome occurs when the clinicians do not present a positive behaviour towards the guidelines (the variable “Positive clinician’s behaviour towards guidelines and clinical protocols” is negated and it is also a core condition) and they do not perceive the usefulness of the INTERCheckWEB (also this variable is negated and a core condition) even if they perceive its ease of use (the variable is present and is a core condition). Concerning the variables related to the patient, this configuration highlights that the patient’s BMI is present as a core condition and, even if as peripheral conditions, there is the presence of all other variables (Patient’s autonomy, Quality of available patient’s data, Patient’s Polytherapy, Patient’s Comorbidities).

**Figure 2:**
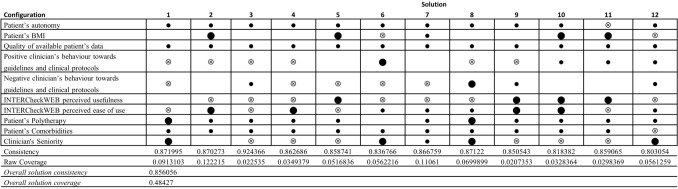
The consistent and empirically relevant configurations.

The results of the whole solution show that the variables related to the patient are present in the most cases (Fig. 2): Patient’s Polytherapy in 11 out of 12; Patient’s Comorbidities and Patient’s autonomy in 11 out of 12 and it is negated in 1 out of 11. Patient’s BMI in 5 out of 12 and it is negated in 2 out of 12. The negation and the presence of the variables related to guidelines and INTERCheckWEB are reported. Some patterns present the negation, or the absence of the variable related to the clinician’s behaviour towards guidelines (5 out of 12). Instead, the “INTERCheckWEB perceived usefulness” is negated in 7 out of 12 patterns. However, when the variable is present, it is a core condition (patterns 5, 9, 10, 11) and in all these patterns the variable Clinician's Seniority is negated, whereas the “INTERCheckWEB perceived ease of use” is negated in 2 out of 4 patterns, and it is present in the other 2 out the four patterns. However, the presence of “INTERCheckWEB perceived ease of use” is present in patterns in which the Clinician's Seniority variable is present or negated. Lastly, the Clinician's Seniority variable is negated in 5 out of 12 patterns and it is present in 5 out of 12 ones, but in 4 out of these 5 patterns is core.

## Discussion

The present study investigated the setting of hospitalized patients, since approximately 45% of hospitalized older patients are discharged with five or more medications^79,80^, and this problem is particularly relevant in Internal Medicine hospitalized patients. In this view, it emerged that the clinical choice to change therapy is becoming relevant to decrease the incidence of drug-related adverse events and improve the adherence to medications, reducing the economic burden on the patients as well as on the healthcare providers^7^. Despite the availability of many tools to minimize drug therapy-related problems, supporting deprescribing activities, there is little guidance for the process of deprescribing, in general clinical practice^81–83^.

One of the most important assets used in deprescribing activities would leverage on digital solutions and guidelines, thus assisting the decision-making process limiting clinicians’ judgement.

According to this consideration, we tried to answer to the presented RQ1, “Which are the factors determining the change in the treatment options in frail patients in polytherapy?”, thus revealing that digital solution perceived ease of use, as well as clinicians’ seniority, number of drugs assumed by the patients and the number of chronic diseases, are the factors that most influence the change in the treatment options in frail patients.

In the investigation of the potential factors impacting on the propensity of the clinician to change the current patients’ therapy (with a deprescribing approach), it emerged that having an IT system easy to use would be a facilitator to the clinical choice. Another important potentially disruptive factor for the introduction of INTERCheckWEB, is related to the sustainability of the digital solution: it is free, no representing a cost for the hospital, and without any organizational factor potentially limiting its hospital adoption. Conversely, deprescribing activities, as well as the use of specific supporting tools, are strictly related to individual clinicians’ factors guiding the clinical choice and managing such frail and chronic patients.

**Figure 3:**
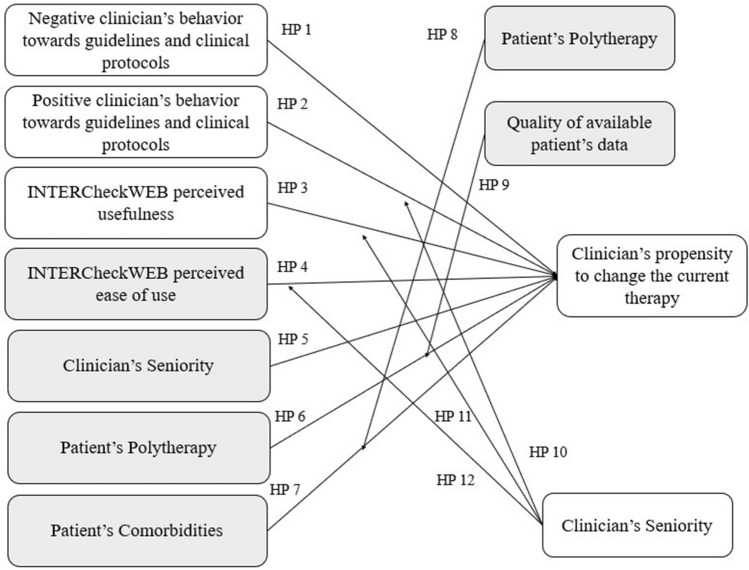
Framework with verified hypotheses.

Furthermore, the number of drugs and the number of concomitant diseases affecting a patient, play a key role in the potential current therapies’ changes (Fig. 3), decreasing the prescription of potentially inappropriate medicant as revealed in other studies^84^.

Based on these results, we tried to highlight “how the combination of inclination of clinicians towards specific information deriving from CPSS INTERCheckWEB and guidelines and other factors affects the change in the treatment options in frail patients in polytherapy?” (RQ2). This analysis confirms that the clinical conditions of the patients are the driving factors that prompts the clinicians towards a therapies’ change. Mainly the patient's comorbidities and drugs taken in combination with other characteristics such as autonomy and BMI as well as the quality of the data.

This result puts in evidence that the deprescribing is a pivotal activity above all for a particular set of patients, characterised by comorbidities and a high number of drugs administered, but the clinicians’ propensity towards the change therapies depends also on the availability of the other patient’s information (BMI, autonomy, quality of data).

The guidelines and the perceived usefulness of INTERCheckWEB are not driving factors; indeed their negation can determine the change of therapies. On this aspect, a deeper analysis should be performed to understand the resistance of clinicians towards the use of INTERCheckWEB, as they do not perceive its usefulness. However, the easy use of INTERCheckWEB is undoubtedly an aspect that could facilitate its daily and routine use, and this do not depend on clinicians’ seniority.

According to these findings, the decision-making process of deprescribing would be based, above all, on patient complexity whereas the individual characteristics plays a minor role, as also declared within the “personalized medicine era”^85^. Thus, possible leverages that the organization could apply to support the decision-making process (guidelines and INTERCheckWEB), do not emerge as relevant for determining the clinicians’ propensity to change the therapy.

The results of the study would enlarge the stream of literature related to the deprescribing activities and tools, that are becoming relevant topics in the care of older adults living with multiple concomitant diseases and assuming multiple medications, thus also including frail conditions in this COVID era, strengthening the need for the clinicians to modify the therapies and to pay attention to the DDIs^16,17,86^. Focusing on COVID-19 disease, the prescription decision-making process would consider the unique aspects of such virus, including the limited treatment options, use of off-label medications, need for rapid decision-making, and increased risk of drug shortages. Healthcare providers need to carefully evaluate the risks and benefits of each medication and monitor patients closely for potential DDIs^87,88^.

The increase in available treatments and the use of single disease model guidelines have led to a healthcare system geared towards prescribing, with deprescribing often seen as a separate activity. Deprescribing should be considered as a part of prescribing and is a key element in ensuring patients remain on the most appropriate medications at the correct doses for them^89,90^.

From a clinical point of view, deprescribing requires a comprehensive review of risk and benefits of a medication in the context of the quality of remaining life, patient and family priorities and preferences. More than 90% of inpatients are taking at least one inappropriate medication and up to 43% of medications taken by older patients lack a clear indication. Moreover, from 5 to 11% of medications may be unintentionally prescribed for the same indication^89^. In this view, polypharmacy increases the risk of adverse drug reactions and hospitalization in elderly: according to this consideration, rational deprescribing of medications, such as anticholinergics, benzodiazepines, antipsychotics, opioids, and proton pump inhibitors, in selected patients may be a good first step to reducing this risk, as well as the occurrence of DDIs^91^.

From a practitioner perspective, deprescribing still remains an activity for which guidelines and digital tools are not perceived by clinicians as useful decision-making support. Therefore, while on the one hand these CPSS are developing in the clinical context, on the other hand there is a lack of full acceptance and understanding of their usefulness on the part of clinicians. Future research will be addressed to define the resistances of the clinicians towards the CPSS and fostering the trust of clinicians in CPSS which is not to be perceived as a substitute to their knowledge but a tool that allows the clinicians to support their decision-making process in prescribing more appropriate therapies for complex patients.

## Ethical approval

The study was approved by the Healthcare Directorates of the hospitals involved (ASST Ovest Milanese, composed of four different hospitals – Legnano Hospital, Magenta Hospital, Abbiategrasso Hospital and Cuggiono Hospital), according to the study protocol number 5135 (Class 03.08.01) dated 8th February 2019. After having received the approval, all methods used for the achievement of the study objective, were carried out in accordance with relevant guidelines and regulations.

### Informed consent

The informed consent was obtained by all the clinicians involved, that were aware that the present research activity had the main aim to publish the results, and they gave their consents in disseminating their perceptions, in an aggregated and anonymous manner.

## Data Availability

All the output data reported in the paper is available upon request, by directly contacting the corresponding authors.
